# Effects of a Community Health Worker–Led Health Literacy Intervention on Lifestyle Modification Among Patients With Hypertension and Diabetes in the City of Harare, Zimbabwe: Protocol for a Cluster Randomized Controlled Trial

**DOI:** 10.2196/47512

**Published:** 2023-10-03

**Authors:** Nyaradzai Arster Katena, Shepherd Shamu, Golden Tafadzwa Fana, Simbarashe Rusakaniko

**Affiliations:** 1 Family Medicine, Global and Public Health Unit Department of Primary Health Care Sciences University of Zimbabwe Harare Zimbabwe; 2 Internal Medicine Unit Department of Primary Health Care Sciences University of Zimbabwe Harare Zimbabwe

**Keywords:** hypertension, diabetes, community health worker, lifestyle modification, cluster randomized controlled trial, protocol, Harare, Zimbabwe, RCT, randomized controlled trial, hypertensive, blood pressure, CHW, diabetic, Africa, healthy lifestyle, health literacy

## Abstract

**Background:**

Hypertension and diabetes are among the most common noncommunicable diseases in Zimbabwe. These 2 conditions are largely prevented and controlled by lifestyle modifications, such as healthy eating habits, physical activity, and avoiding the use of tobacco and alcohol.

**Objective:**

This study aims to evaluate the effects of a community health worker (CHW)–led health literacy intervention on lifestyle modification among patients with diabetes and hypertension in the City of Harare, Zimbabwe, in terms of (1) adherence to recommended lifestyle changes, (2) blood pressure and blood glucose levels, and (3) medication adherence.

**Methods:**

This is a 6-month–long cluster randomized controlled trial, which will be conducted in primary health care clinics in the city of Harare. A total of 14 clinics will be randomly allocated into either the intervention or control group. A total of 680 (49 participants per clinic) adult patients with hypertension and diabetes will be enrolled for the trial. Participants in the intervention arm will receive a simple health literacy intervention on lifestyle modification, which will be delivered by trained CHWs, while those in the control arm will receive usual care. The intervention consists of 4 face-to-face interactive educational sessions and monthly support visits by trained CHWs and will be carried out over a period of 6 months. The primary study outcomes will be blood pressure and blood glucose levels and levels of adherence to the recommended lifestyle modifications. Other outcomes will include adherence to medication. Data management and analysis will be done using Epi Info software, and the data will be analyzed using the intention-to-treat principle.

**Results:**

Recruitment of study participants commenced on June 1, 2023, and was completed on July 30, 2023. Baseline data are being collected after participants’ recruitment, and the final data collection will be completed by January 31, 2024.

**Conclusions:**

There is an information dearth regarding the effectiveness of CHW-led interventions for the management and control of hypertension in Zimbabwe. Our study offers an opportunity to show the effectiveness of CHWs in addressing the gaps in the management of hypertension and diabetes. The findings from our study will provide crucial evidence on the effectiveness and feasibility of a simple intervention delivered by CHWs in resource-constrained areas.

**International Registered Report Identifier (IRRID):**

PRR1-10.2196/47512

## Introduction

Hypertension and diabetes are among the leading causes of morbidity, disability, and premature death worldwide. In 2019, diabetes was the direct cause of 1.5 million deaths worldwide, and 48% of all deaths due to diabetes occurred before the age of 70 years [1-3]. An estimated 1.28 billion adults aged 30-79 years worldwide have hypertension, with an estimated 46% being unaware of their condition. The prevalence of both hypertension and diabetes has been increasing more rapidly in low- and middle-income countries, compared to high-income countries. This is a cause for concern in these countries, considering their already high burden of communicable diseases [1,3].

Studies have shown links between hypertension and diabetes [4-6]. For instance, hypertension is more common in patients with diabetes, and similarly, diabetes is also more common in patients with hypertension than in the general population [4]. Previous studies have reported that hypertension occurs in 50% to 80% of patients with type 2 diabetes [5]. These two conditions are predominantly lifestyle related, as they are highly associated with risk factors like obesity, stress, excessive salt intake, physical inactivity, excessive alcohol intake, and smoking [3,6,7]. Therefore, prevention, control, and management of these two conditions involve lifestyle modifications. The recommended lifestyle changes include weight reduction, healthy eating, reducing alcohol consumption, regular physical exercise, and quitting smoking [8-10].

Lifestyle modification poses a challenge for patients with hypertension and diabetes, and several studies have demonstrated high noncompliance with the recommended lifestyle modifications worldwide [11]. Although studies have shown that nonadherence to recommended lifestyle modifications is influenced by several factors, inadequate knowledge and lack of support have constantly been highlighted as major contributing factors [12-17]. Therefore, there is a need for educational interventions to assisst patients with hypertension and diabetes in adopting the reccommended lifestyle modifications easily. In most instances, counseling about the reccommended lifestyle modification is done by physicians and nurses as part of their routine clinical care [14]. However, in resource-limited settings, where there is a shortage of health care workers, effectively counseling patients on the reccommended lifestyle modifications poses a challenge [18]. As a result, there is a need to explore other cost-effective strategies to ensure the effective counseling of patients regarding lifestyle modifications. Task-shifting to community health workers (CHWs) has been proven as one such cost-effective and sustainable approach in chronic disease prevention and control, particularly in areas with limited economic and health care resources [15,16,18].

CHWs are voluntary public health workers who are trusted members of the community they serve [17]. They play a crucial role in primary and tertiary prevention of diseases, particularly in low- and medium-income countries. According to the Alma Ata Declaration on Primary Health Care, CHWs are the cornerstone of health promotion, as they facilitate community members’ participation in health programs [19]. Overall, CHWs build individual and community capacity to manage chronic conditions by increasing health knowledge and self-sufficiency through a range of activities, such as outreach, community education, informal counseling, social support, and advocacy [20-22]. In Zimbabwe, CHWs primarily provide community-based care, but for many years, their efforts have been focused on home-based care for people living with HIV and tuberculosis, with limited focus on addressing noncommunicable diseases (NCDs) [21]. This shows the need to continuously come up with effective CHW-led interventions for the management of common NCDs.

We have developed a simple CHW health literacy intervention on lifestyle modification, and we have designed a study to evaluate the effects of the intervention among patients with hypertension and diabetes in the city of Harare, Zimbabwe. This paper describes our research design and discusses the rationale of our study. We hypothesize that the intervention will have a positive effect on (1) adherence to recommended lifestyle modifications, (2) adherence to medication, as well as (3) blood glucose and blood pressure levels.

## Methods

### Study Design

The study is a cluster randomized controlled trial, which will be conducted in the city of Harare in Zimbabwe from June 2023 to January 2024. The design, implementation, and reporting of the study are based on the recommendations from CONSORT (Consolidated Standards of Reporting Trials) statement on cluster randomized controlled trials [23]. The cluster randomized controlled trial will have a 6-month follow-up period and will consist of 1 intervention arm and 1 control arm.

### Study Setting

The study will be conducted in 14 primary health care clinics in the city of Harare; these clinics were purposefully selected from a total of 39 health facilities (12 polyclinics, 6 family health services clinics, 15 satellite clinics, and 2 infectious diseases hospitals) in the city. The 14 facilities that have been selected for the study are the ones that offer comprehensive services, including curative services, chronic pain care, maternal and child health services, HIV prevention services, and community health services. These clinics are in the high-density suburbs of the city, with each clinic serving a population of 80,000-100,000 people, the majority of whom are socioeconomically disadvantaged. The 14 health facilities will be randomly allocated into either the intervention or the control arm. Thus, each study arm will comprise 7 health facilities.

### Interventions

The intervention arm will receive the CHW-led health literacy intervention, while the control arm will receive usual care.

#### CHW-Led Health Literacy Intervention for Lifestyle Modifications

The intervention was designed by the principal investigator (PI), and its development entailed two processes, as follows: (1) conducting a scoping review of the literature on CHW-based interventions for hypertension and diabetes and (2) validating the content through consultations with 10 experts (ie, 3 health promotion officers, 2 nutritionists, 2 medical doctors, 1 Diabetes Association of Zimbabwe official, 1 pharmacist, and 1 nurse). The intervention is based on the constructs of the Health Belief Model [24], and thus, it aims to (1) convey the consequences of the patient’s poor adherence to recommended lifestyle modifications, (2) communicate to the patient the list of recommended lifestyle modifications and highlight the benefits, (3) provide assistance in identifying and reducing barriers to adopt the recommended lifestyle modifications, and (4) develop cues to action for the patient.

This intervention consists of face-to-face educational sessions and support visits to patients and will be delivered by trained CHWs. A total of 4 educational sessions will be conducted at either the patient’s home or the primary care clinic, through which the patient is routinely monitored. The first session will be held at the clinic on the day of recruitment. The second and the third sessions will be held at either the clinic or the patient’s home, as preferred by the patient. The fourth session will be a home visit, where the CHW will visit the patient in their home to provide support and follow-up. After the fourth educational session, the CHWs will continue to make monthly home visits to the patient.

In terms of content, in the first session, the CHW will educate the patient on the recommended lifestyle modifications and the consequences of nonadherence. In the second session, focus will be on the patient’s barriers to adopting recommended lifestyle modifications and ways to overcome them. At the end of this session, patients are expected to write down their own plan for lifestyle modifications on a card, which they will then place in a visible location in their home. This will serve as a cue to action. The second session will be highly interactive, compared to the first session. The third and fourth sessions will be supportive and evaluation sessions during which the CHW assesses whether the patient has been following the action plan established in the second session. In the fourth session, which will be conducted at home, available family members will be involved to support the patient, and this will serve as another cue to action for the patient and will also help to build the patient’s self-efficacy. Any deviations from the plan will be discussed and ways to correct will be explored. The monthly home visits conducted after the fourth educational session will serve as support visits, where the CHW will continue to reinforce the importance of adhering to lifestyle modifications.

In terms of timing, the first session will be conducted on the day of recruitment, and the second session will be scheduled a week after the first session. The third session will be conducted 2 weeks after the second session, and the fourth session will also be conducted 2 weeks after the third session. A month after the fourth session, the patients will receive monthly home visits at a date and time convenient for them. Each educational session will last 45-60 minutes, while the monthly support visits will last 10-15 minutes.

To ensure that the intervention adheres to the protocol and maintains study fidelity, the CHWs who will deliver educational sessions will undergo a 1-day training; this training will be conducted by the PI, 1 nurse, and 1 health promotion officer from the city of Harare. The CHWs will be given notes on the recommended lifestyle modifications, to which they will refer when conducting educational sessions. All the CHWs participating in the study have been working as CHWs for periods ranging from 5 to 30 years, and upon their initial recruitment as CHWs, they underwent an 8-week training program. They will also receive refresher trainings as needed, in accordance with the guidelines for CHWs in Zimbabwe [21,25].

#### Usual Care

The usual care for patients with diabetes and hypertension in the city of Harare involves patients coming to the clinic for monthly reviews and medication collection. Patients also receive counseling on medication adherence and recommended lifestyle modifications, particularly on the day when they are diagnosed and start their medication. Although there is no formalized CHW intervention, CHWs are involved in the continuum of care when patients fail to report for their monthly reviews for 3 consecutive months. Additionally, patients may seek consultation with CHWs in their communities, as needed. Table 1 summarizes the interventions that will be implemented in this study.

**Table 1:** Summary of study interventions.

Component	Control arm (usual care)	Intervention arm (community health worker–led health literacy intervention)
Main components	Health education session on the importance of adhering to medication and lifestyle modifications on the day medication is initiated by a nurse or doctor and monthly drug pickups at local health facilities	Usual care and 4 interactive educational sessions and monthly home visits by community health workers
Theoretical background of educational sessions	None	Health Belief Model [24]
Role of community health workers	Follow-up when the patient misses clinic appointments for 3 consecutive months	Continuous support in the management and control of diabetes and hypertension

#### Randomization

All 14 targeted health facilities will be randomly allocated to either the intervention or the control arm. Each arm will consist of 7 clinics. The randomization process will be conducted by an official from the city of Harare, from the City Health Department, who will not be part of the study staff. The randomization process will be done using the simple randomization technique, based on computer-generated random numbers. To reduce bias and improve study fidelity, group allocation will be concealed to the data collectors until the completion of baseline data collection.

#### Inclusion and Exclusion Criteria

All adults aged 18 years and older, with a confirmed diagnosis of hypertension and diabetes, residing within the targeted health facility’s catchment area, and capable of providing written informed consent will be eligible for enrollment.

Exclusion criteria will include the following: (1) individuals having hypertension- and diabetes-related complications that cannot be managed in a primary care setting, (2) individuals who are pregnant or planning to become pregnant during the course of the trial, (3) individuals who will not be able to stay within the catchment area of the clinic where they are enrolled for the duration of the trial, and (4) individuals who will not be able to give consent for any reason, such as severe mental health disorders.

#### Recruitment of Participants

Participants will be recruited by trained research assistants every day of the 1-week recruitment period. The recruitment process will be done concurrently at control and intervention sites. Baseline data will be collected on the day of the recruitment, and research assistants will not be aware of group allocations during the recruitment and the baseline data collection periods.

#### Outcome Measures

The outcome evaluation for this study will be based on data collected both at baseline and during the postintervention follow-up (after 6 months). Data collection tools will be identical for data collection at both periods and include information on patient’s demographics, disease history, lifestyle behaviors, medication use, primary use of health care services, and hospitalizations. Height, weight, waist circumference, glycosylated blood glucose levels, and blood pressure will also be measured.

The primary study outcomes will be blood pressure and blood glucose levels as well as levels of adherence to recommended lifestyle modifications. Other outcomes will include changes in medication adherence and additional health-related outcomes, such as frequency of hospitalization or the occurrence of complications.

#### Assessment of Primary and Secondary Outcomes

A consolidated interviewer-administered questioner comprising of all measures (as detailed in Multimedia Appendix 1) will be used to collect data. The interviewer-administered questionnaire consists of 5 sections, as follows: section A (sociodemographic characteristics), section B (details of the patient’s condition), section C (blood pressure, BMI, and glycosylated hemoglobin [HBA_1c_] measurements), section D (adherence to lifestyle modifications), and section E (adherence to medication).

##### Lifestyle Modifications

Patients’ levels of adherence to recommended lifestyle modifications will be assessed using self-reported questions adapted from the Hypertension Self-Care Activity Level Effects Scale (H-SCALE) questionnaire. The H-SCALE is a validated self-reported questionnaire for assessing self-care practices for hypertension prevention and control [26]. The questionnaire assesses 5 key recommended lifestyle modifications (ie, low-salt diet, physical activity, smoking cessation, weight management, and abstinence from alcohol) as well as adherence to medication. Table 2 shows how each recommended lifestyle modification will be assessed based on the H-SCALE.

**Table 2:** Assessment of lifestyle modifications using an interviewer-administered questionnaire adapted from the Hypertension Self-Care Activity Level Effects Scale (H-SCALE).

Recommended lifestyle modification	The WHO^a^ recommendation	Assessment method
Weight reduction and management	Maintain normal body weight (BMI 18.5-24.9 kg/m^2^)	First, the patient’s BMI will be assessed based on the patient’s height and weight. All patients with a BMI above 24.9 will be considered as overweight. Second, 10 questions will be used to assess activities undertaken to manage weight through dietary practices, such as reducing portion size, making food substitutions, and exercising to lose weight. Participants who say yes to 5 or more questions will be nonadherent to recommended weight management practices.
Reducing dietary salt intake	Reduce dietary salt intake to less than 2400 mg of sodium per day or less than 6000 mg sodium chloride per day.	A total of 6 questions will be used to assess practices related avoiding salt while cooking and avoiding foods high in salt content. Patients who answer yes to 3 or more questions will be considered as nonadherent to a low salt diet.
Reducing dietary fat intake	Avoid foods high in cholesterol and fat content	A total of 2 questions will be used to assess practices related to avoiding foods high in cholesterol and fat content. Patients who answer yes to any 1 question will be considered as nonadherent to a low fat or cholesterol diet.
Increasing fruit and vegetable consumption	Consume 5 servings (equivalent of 5 tablespoons) of vegetables and 3 servings (equivalent of to at least 3 fruits) of fruits every day.	A total of 2 questions will be asked to assess adherence to fruits and vegetables. Patients who answer yes to any 1 question will be considered as adherent to a high fruit and vegetable diet.
Smoking	Avoid smoking	Smoking status will be assessed by 1 question. All patients who answer yes to the question will be considered nonadherent to nonsmoking recommendations
Moderation in alcohol consumption	Limit alcohol consumption to no more than 2 drinks (eg, 24 oz of beer, 10 oz of wine, or 3 oz of 80-proof whiskey) per day in men and no more than 1 drink per day in women and lighter-weight persons.	Alcohol intake will be assessed using 2 questions. Participants who answer yes to any 1 of the questions will be considered nonadherent to the alcohol recommendation moderation.
Physical activity	Engage in regular aerobic exercises, such as brisk walking for at least 30 minutes per day, for at least 5 days a week.	Physical activity will be assessed by 1 question. Participants who answer yes to the question will be considered as being adherent to the physical activity recommendation.

^a^WHO: World Health Organization.

##### Hypertension and Diabetes Control

Patient’s hypertension and diabetes control will be assessed by blood pressure and HbA_1c_ measurements, respectively*.* Blood pressure of 140/90 mm Hg or higher will be considered uncontrolled hypertension, while an HbA_1c_ level above 8% (53 mmol/mol) will be considered uncontrolled diabetes [27].

##### Adherence to Medication

Patients’ adherence to medication will be assessed using a widely used and validated tool, the 8-item Morisky Medication Adherence Scale (MMAS-8) [28-31]. The MMAS-8 consists of 8 questions that assess patients’ adherence to medication. Patients who score 8 points on the MMAS-8 will be considered to have high adherence, and patients who score 7 and 6 will be considered to have medium adherence; those scoring less than 6 points will be classified as having low adherence.

##### Other Health Outcomes

Other health outcomes, such as hospitalization and the occurrence of diabetes and hypertension complications, will be assessed from the patients’ clinic records*.*

##### Sample Size

Based on the recommendations for sample size calculation for cluster randomized controlled trials with a fixed number of clusters [32], a total of 680 participants will be required for the trial. Therefore, the study will comprise 14 clusters, with 49 participants per cluster. The sample size was calculated using the following parameters, obtained from the review of literature: 80% study power, 0.5 effect size, 1 SD, 0.05 coefficient of variation, and 20% lost to follow-up adjustment.

##### Data Collection and Management

Recruitment of participants started in June 2023. Baseline data were collected during the recruitment of participants. Follow-up data will be collected after 6 months, in January 2024. Data will be collected using the research assistants’ smartphones. The questionnaire will be uploaded onto the phones and the REDCap platform will be used for storing and managing the data. The computer system that will be used for storing the data will be password protected so that the data will only be accessible to authorized individuals. Patients will be assigned unique identifying numbers at the beginning of the study and the same number will be used in the follow-up data collection period. Data will be managed according to the International Conference on Harmonisation Guidelines for Good Clinical Practices [33]. To minimize errors, range checks and skip patterns within data entry screens will be used. Data will be backed up on a password-protected external hard drive as well as on Google Drive.

#### Data Analysis

Data analysis will be performed using the Epi Info (Centers for Disease Control and Prevention) software. The primary analysis will be conducted under the intention-to-treat principle. For individual outcomes, proportions will be compared using the chi-square test, and continuous measures will be compared using 2-tailed *t* tests. Stratified analysis will be conducted to control for confounding and assess the effect modification by demographic variables and medication adherence. The effects of the intervention will be tested by ANOVA, which will allow adjustment for baseline differences between groups. Two-sided *P* values and 95% CIs will be used to determine statistical significance of results.

#### Ethical Considerations

Permission to carry out the study in the city of Harare clinics was obtained from the City Health Services Director. Ethical clearance was obtained from the University of Zimbabwe’s institutional review board, the Joint Research and Ethics Committee (JREC/339/2022). Written informed consent will be obtained from all study participants. Confidentiality of the study participants will be maintained at all stages of the study.

All participants will be treated fairly, and they will not be intentionally subjected to any harm. During the study, all participants who present with any other health education needs that do not result in contamination of the study will be assisted. Those with complications or any other needs beyond the scope of the research team will be referred to appropriate HCWs. The knowledge that will be generated from this study will be disseminated to all pertinent stakeholders using various strategies, such as publications and feedback meetings.

## Results

Recruitment of study participants commenced on June 1, 2023, and was completed on July 30, 2023. Baseline data are being collected after participant recruitment, and the final data collection will be completed by January 31, 2024.

## Discussion

### Expected Findings

Hypertension and diabetes are global health challenges that have placed significant burdens on health care systems in all nations [34]. The burden of chronic NCDs is rapidly increasing worldwide. Nevertheless, many countries, particularly low- and medium-income countries, do not have adequate health care resources to effectively deal with these prevalent health problems. Therefore, there is a dire need for effective interventions to tackle these challenges. These interventions rely on adequate human resources. However, in resource-limited countries like Zimbabwe, there is a shortage of health workers, and they are already overburdened with the management of infectious diseases, such as HIV or AIDS, tuberculosis, and diarrheal diseases. Therefore, task-shifting the counseling responsibilities for patients with chronic NCDs is one of the options that should be considered. The findings from our study will thus add to the body of knowledge needed for the development of interventions to address NCDs in resource-limited settings.

Evidence-based national and international guidelines on managing hypertension and diabetes are well-established, cost-effective approaches, suitable for adoption in resource-constrained settings, but they have not been adequately investigated [8]. Our study offers an opportunity to show the effectiveness of CHWs in addressing the gaps in the management of hypertension and diabetes. The use of CHWs to address the shortage of professional health care workers in the management of hypertension, diabetes, and other chronic NCDs has been widely recommended [35]. Evidence from several low- and medium-income countries indicates that this is a feasible and cost-effective strategy [36-38]. However, there is an information dearth regarding such evidence, specifically for the Zimbabwean setting, and particularly, in the urban setup. To the best of our knowledge, our study will be the first to assess the effectiveness of CHWs in improving the much-needed lifestyle modification among patients with hypertension and diabetes in the city of Harare. The findings from our study will provide evidence on the feasibility and effectiveness of a simple intervention delivered by the CHWs in resource-constrained areas. Overall, our study is vital to the achievement of Sustainable Development Goal target 3.4, which aims to reduce premature mortality from NCDs by one-third [39].

### Strengths and Limitations

The strengths of our study include CHWs who are already integrated in the Zimbabwean primary health care system. The CHWs have prior experience in disseminating health education on various health topics, particularly prevention of diarrheal diseases and malaria, which are prevalent in the country. Additionally, the CHWs are routinely involved in follow-ups with patients who have hypertension and diabetes, ensuring they do not miss monthly checkups. Therefore, if successful, our intervention will modify and strengthen community-based management of hypertension and diabetes. Unlike most of the CHW-based interventions being implemented in other low- and middle-income countries, our intervention is based on a tested and tried health behavior model, the Health Belief Model [24]. Therefore, we envisage that the intended behavior change in the adoption of recommended lifestyles will be successful. Moreover, our intervention will be innovatively addressing some of the most challenging barriers to the management of chronic NCDs, such as human resources. Hence, if successful, this intervention can be potentially scaled up to the entire nation.

Nonetheless, there are weaknesses in our study, mainly emanating from the cluster randomized controlled trial design. First, our study does not involve blinding of study participants. Lack of blinding is one of the weaknesses inherent in cluster randomized controlled trials that include behavioral interventions. However, to reduce bias associated with lack of blinding, there will be partial blinding of data collectors during the collection of baseline data. The data collectors will not know the arm to which each clinic will belong. In addition, there is a possibility of information sharing among participants in the intervention and control group, since the suburbs in Harare are within close proximities. To limit the sharing of information, participants in the intervention group will receive a detailed explanation of the study’s importance from the PI. There is also a possibility of social desirability bias in assessing lifestyle modifications. Participants may report what they perceive as the desired behavior rather than their actual behavior. However, the study will assess overall changes in blood glucose and hypertension levels as well as BMI, which are more objective outcome measures.

### Conclusions

Our trial aims to evaluate the effects of a CHW-led health literacy intervention for lifestyle modifications among patients with diabetes and hypertension in Harare, Zimbabwe. Findings from our study will provide reliable evidence on the effectiveness of a simple community-based intervention in the management and control of the two most common NCDs in Zimbabwe. In summary, our study will contribute to the much-needed body of knowledge on the effectiveness of using CHWs in the management of chronic NCDs in resource-limited countries.

## Appendix

Multimedia Appendix 1 Interviewer-administered questionnaire for collecting data.

## Abbreviations

CHW: community health worker
CONSORT: Consolidated Standards of Reporting Trials
H-SCALE: Hypertension Self-Care Activity Level Effects Scale
HBA_1c_: glycosylated hemoglobin
MMAS-8: Morisky Medication Adherence Scale-8
NCD: noncommunicable disease
PI: principal investigator

## Data Availability

The consent and ethical approvals for this study do not have any restrictions on data sharing. Therefore, the data sets generated from this study will be made publicly available as supplementary files in all publications of the study results.
