# Supplementary material for: Effects of a Community Health Worker–Led Health Literacy Intervention on Lifestyle Modification Among Patients With Hypertension and Diabetes in the City of Harare, Zimbabwe: Protocol for a Cluster Randomized Controlled Trial
Source: JMIR Res Protoc. 2023 Oct 3;12:e47512. doi: 10.2196/47512 (PMC10582805; doi:10.2196/47512)
Supplement: Multimedia Appendix 1 [file resprot_v12i1e47512_app1.doc]

| |  | | --- |   **Date………………………………… Patient Number**  **Data Collection Period Baseline Follow up**  **Site: ……………………………………….**  **Name of data Collector** ……………………………… |
| --- | --- |

| **Section A: Socio-demographic details** | |  |
| --- | --- | --- |
| A1: Age (years) | | ____________ |
| A2: Sex | | 1. Male 2. Female |
| A3: Place of residence | | ____________ |
| A4: Marital status | | 1. Single 2. Married 3. Divorced 4. Widowed 5. Co-habiting |
| A5: Highest level of education | | 1. Never been to school 2. Primary 3. Secondary 4. Tertiary |
| A6: Employment status | | 1. Informal employment 2. Formal employment 3. Not employed |
| A7: Religion | | 1. Christian 2. Moslem 3. Traditionalist 4. Apostolic 5. None 6. Other specify______________________ |
| **SECTION B: CONDITION DETAILS** | | |
| B1: Condition | | - 1. Diabetes   2. Hypertension   3. Both |
| B2: Medication being taken | | …………………………………… |
| B3: Duration of illness | | ……………………………………. |
|  | |  |
| **SECTION C: Assessment of Overall Health Status- Data collector verify the patient’s report with clinic card** | | |
| C1: Has the patient been admitted due to problems associated with hypertension/diabetes during the period under review (probe for details about the admission) | | - - 1. Yes     2. No   Details…………………………………….. |
| C2: Has the patient experienced any complications due to hypertension/ diabetes during the period under review (probe for details about the complications) | | - - 1. Yes     2. No   Details…………………………………… |
| **SECTION D: MEASUREMENTS** | | |
| B1: Height (m) |  | |
| B2: Weight (Kg) |  | |
| B3: BMI ( weight- Kg)/ (height- m)^2^ |  | |
| B4: BP: |  | |
| B5: HBA1c level |  | |
| **Section D: Assessment of lifestyle modification** | | |
| **D1:Adeherence to low salt diet:**  ***In the past 7 days did you…***   - Eat potato chips, salted nuts, or salted popcorn? Yes .............. No............. - Eat processed meats such as ham, bacon, bologna, or sausage? Yes........... No.............. - Eat smoked meats or smoked fish? Yes........... No............ - Eat fast foods like pizza, potato crisps and pies? Yes......... No.............. - Add salt your food at the table? Yes.......... No.............. - Add salt to food when you’re cooking? Yes......... No..............   **SCORE (total number of Yes)**  **D2:Adeherence to low fat diet:**  ***In the past 7 days did you…***   - Eat fried foods such as fried chicken, french fries, or fish? Yes......... No.............. - Eat any other fatty foods? Yes......... No..............   **SCORE (total number of Yes)**  **D3: Adherence to fruit and vegetable consumption**  ***In the past 7 days did you....***  Consume five servings (equivalent of 5 table spoons) of vegetables for all the 7 days  Yes......... No..............  Consume three servings (equivalent of to at least any three fruits) of fruits every day  Yes......... No..............  **SCORE (total number of Yes)**  **D4: Alcohol consumption**  *A drink* of alcohol is defined as: one 12-oz can or bottle of beer, one 4-oz glass of wine, one 12-oz can or bottle of wine cooler, 1 mixed drink or cocktail, or 1 shot of hard liquor.  On a typical day that you drink alcohol, how many drinks do you have?  (yes) More than 2.......... (No) not more than 2.......  How many days do you drink in a typical week? (yes) more than 5................ (no) not more than 5......................  **SCORE (total number of Yes)**  **D5: Smoking**  Do you currently smoke cigarettes or sniif any other type of tobacco? Yes............ No................**.**  **SCORE (total number of Yes)**  **D6 : Physical Activity**  In the last 7 days, did you exercise for at least 30 minutes per day fo five or more days? Yes....... No.............  **SCORE (total number of Yes)**  **D7: Weight management practices**  ***As part of day to day living, in order to lose weight or maintain my weight***…   - I am careful about what I eat. Yes......... No.............. - I read food labels when I grocery shop. Yes......... No.............. - I exercise in order to lose or maintain weight. Yes......... No.............. - I have cut out drinking sugary sodas and sweet tea. Yes......... No.............. - I eat smaller portions or eat fewer portions. Yes......... No.............. - I have stopped buying or bringing unhealthy foods into my home. Yes......... No.............. - I have cut out or limit some foods that I like but that are not good for me. Yes......... No.............. - I eat at restaurants or fast food places less often. Yes......... No.............. - I substitute healthier foods for things that I used to eat. Yes......... No.............. - I have modified my recipes when I cook. Yes......... No..............   **SCORE (total number of Yes)** | | |
| **Section E: Adherence to Medication**   - Do you sometimes forget to take your medication?   Yes......... No..............   - People sometimes miss taking their medications for reasons other than forgetting. Over the past two weeks, were there any days when you did not take your medication   Yes......... No..............   - Have you ever cut back or stopped taking your medication without telling your doctor because you felt worse when you took it?   Yes......... No..............   - When you travel or leave home, do you sometimes forget to bring your medication?   Yes......... No..............   - Did you take all your medication yesterday?   Yes......... No..............   - When you feel like your symptoms are under control, do you sometimes stop taking your medication?   Yes......... No..............   - Taking medication is a real inconvenience for some people. Do you ever feel hassled about sticking to your treatment plan?   Yes......... No..............   - How often do you have difficulty remembering to take your medication   Never/ rarely...................  Once in a while................  Sometimes.......................  Usually............................  All the time......................  **SCORE (total number of Yes/ all the time )**  **THE END, THANK YOU!** | | |
